# Microvascular impairment as a biomarker of diabetic retinopathy progression in the long-term follow up in type 1 diabetes

**DOI:** 10.1038/s41598-020-75416-8

**Published:** 2020-10-26

**Authors:** Fabio Scarinci, Fabiana Picconi, Gianni Virgili, Monica Varano, Paola Giorno, Simona Frontoni, Mariacristina Parravano

**Affiliations:** 1 Department of Ophthalmology, IRCCS-Fondazione Bietti, Rome, Italy; 2 Unit of Endocrinology, Diabetes and Metabolism, S. Giovanni Calibita Fatebenefratelli Hospital, Department of Systems Medicine, University of Rome Tor Vergata, Rome, Italy; 3 Department of Neurosciences, Psychology, Drug Research and Child Health (NEUROFARBA), University of Firenze and AOU Careggi, Florence, Italy

**Keywords:** Predictive markers, Diseases

## Abstract

This study aimed to explore differences in vascular and structural parameters using optical coherence tomography angiography in patients with type 1 diabetes (DM1) with mild signs of diabetic retinopathy (DR) over a two-year follow-up period. Parafoveal vessel density (PVD) and foveal avascular zone (FAZ) area were analyzed. The thickness of three predefined retinal slabs was measured, including the inner limiting membrane (ILM)–inner plexiform layer (IPL), IPL–inner nuclear layer (INL), and the IPL–outer nuclear layer (ONL). Twenty-two patients with DM1 and 21 controls were included. There was no significant difference in the FAZ area, perimeter and acircularity index between cohorts over time. Baseline superficial capillary plexus PVD was approximately 10% lower in patients with diabetes than in controls (*p* = 0.001), and was 12% lower at 2 years (*p* = 0.002). There was no difference in the annual linear trend between the groups (− 0.5% in diabetics vs. controls, *p* = 0.736). Baseline deep capillary plexus (DCP) PVD was slightly lower in diabetics than in controls (− 4.4%, *p* = 0.047) and the difference increased at 2 years (− 12.6%, *p* < 0.001). The annual linear trend was − 2.7% in diabetic patients compared to controls (*p* = 0.009)_._ In addition, the PVD of the DCP and the intermediate capillary plexus (ICP) were evaluated separately. Regarding the DCP PVD, no statistically significant difference at any time points in diabetic patients compared to controls and no statistically significant difference in the linear trend was found (*p* > 0.1). Conversely, no difference was recorded for parafoveal ICP density at individual time points (*p* > 0.1), but a statistically significant difference in the linear trend over time in diabetic patients compared to controls was recoded (− 3.2% per year, *p* = 0.001). Despite the apparent intergroup differences at baseline in structural OCT parameters, the differences including ILM–IPL (*p* = 0.273), IPL–INL (*p* = 0.708), and IPL–ONL (*p* = 0.054) were modest and not statistically significant with time. Therefore, the microvascular change of the deeper vessels might be a robust biomarker to evaluate the clinical progression of DR in DM1.

## Introduction

The number of cases of diabetes mellitus is expected to rise to 642 million by 2040. Diabetic retinopathy (DR) remains the most important cause of visual impairment in individuals in several middle-income and high-income countries^1–4^.

Studies have reported a high prevalence of DR of up to 86% in patients with type 1 diabetes mellitus (DM1) in Europe, south Asia, and the USA^5,6^.

Among the several determinants for the development and progression of DR, important risk factors include duration of the disease, poor glycemic control, glycemic variability, genetic elements, hyperlipidemia, and hypertension^5,7–14^.

In addition, puberty has been associated with worsening of DR in patients with DM1^15–17^, Moreover, a study reported that the incidence of DM1 was two times higher in men after puberty^18^.

Using the Early Treatment Diabetic Retinopathy Study DR severity score (DRSS) and color fundus photography, retinal abnormalities in DR are classified into distinct stages based on disease progression^19^. However, microvascular changes appear before clinical signs/findings of DR become evident^20^.

In areas of capillary nonperfusion in DR, capillary occlusions can promote the progression of retinal disease. These microvascular changes can cause angiogenic complications such as proliferative diabetic retinopathy, stimulate the expression of vascular endothelial growth factor, and finally cause breakdown of the blood–retina barrier, which are the major causes of significant DR-related vision loss^3,21,22^.

In light of this, accurate evaluation of capillary nonperfusion or ischemic areas is important to understand the progression of DR and eventually assess the therapeutic outcomes.

Optical coherence tomography angiography (OCTA) is a relatively new tool that can separately visualize and noninvasively quantify retinal capillary layers as well as retinal blood flow in healthy and diabetic eyes^23–26^. In addition, OCTA enables analysis of the superficial and deep capillary plexuses (SCP and DCP, respectively)^27,28^. Moreover, this tool can estimate the enlargement and symmetry of the foveal avascular zone (FAZ), which has been associated with decreased visual function and occurrence of neovascular complications in DR^29,30^.

The mechanisms underlying retinal ischemia in DR remain poorly understood, and there are no OCTA studies showing the vascular alterations in DM1 over time.

Visualization of the thickness of individual retinal layers within the fovea and the perifoveal area, by spectral-domain optical coherence tomography (SD-OCT), has shown thinning of the retinal nerve fiber layer (RNFL) as well as the ganglion cell layer (GCL), and an increase in the inner nuclear layer (INL) in diabetic patients^22,31–36^.

This study aimed to investigate microvascular retinal changes, using automated, quantitative measurements from OCTA data, and structural changes in the selected retinal layers by SD-OCT in DM1 patients diagnosed with mild signs of non-proliferative diabetic retinopathy (NPDR) over a two-year follow-up period.

## Results

Twenty-two patients with DM1 and 21 healthy control subjects were included. There was no significant difference in age (44.9 ± 11.1 years vs. 38.5 ± 11 years, *p* = 0.06) or in the percentage of women (62% vs. 59%, *p* = 0.8) between the DM1 and healthy control cohorts. Patients with DM1 had a mean disease duration of 20.7 ± 10.5 years and an average HbA1c of 7.5 ± 0.9 at baseline, 7.8 ± 0.8 at one-year follow up and 7.6 ± 0.9 at the two-year follow up. All patients were undergoing treatment with basal-bolus insulin, and most of them were under continuous subcutaneous insulin infusion.

Visual acuity in DM1 eyes was 89 ± 4.4 letters at baseline, 87.4 ± 4.4 at one-year follow up, and 87.4 ± 4.1 at the two-year follow up visit, with no significant changes over time.

In the FAZ area, statistically significant differences were not observed at baseline between DM1 eyes and controls (+ 6.9%, *p* = 0.656), as well as at 2 years (+ 4%, *p* = 0.568), based on OCTA analysis of microvascular changes. The annual linear trend did not differ between the groups (− 1.6% in DM1 eyes vs. controls, *p* = 0.737).

At baseline, FAZ perimeter did not show any statistically significant difference between controls and diabetics (log10 values [standard error]: 3.11 [0.77] vs. 3.27 [0.75], *p* = 0.126), and it was also similar at 2 years (3.28 [0.75] vs. 3.27 [0.77], − 1%, *p* = 0.978).

There was no difference in the annual linear trend between the groups (− 16% in diabetics vs. controls, *p* = 0.255). Acircularity index was similar at baseline (+ 1.5% higher) in patients with diabetes and healthy controls (*p* = 0.174), as well as at 2 years (11%, *p* = 0.451). There was no difference in the annual linear trend between the groups (− 1% in diabetics vs. controls, *p* = 0.297).

Baseline SCP parafoveal vessel density (PVD) was approximately 10% lower in diabetics than in controls (*p* = 0.001), and was 12% lower at 2 years (*p* = 0.002). There was no difference in the annual linear trend between the groups (− 0.5% in diabetics vs. controls, *p* = 0.736).

**Figure 1:**
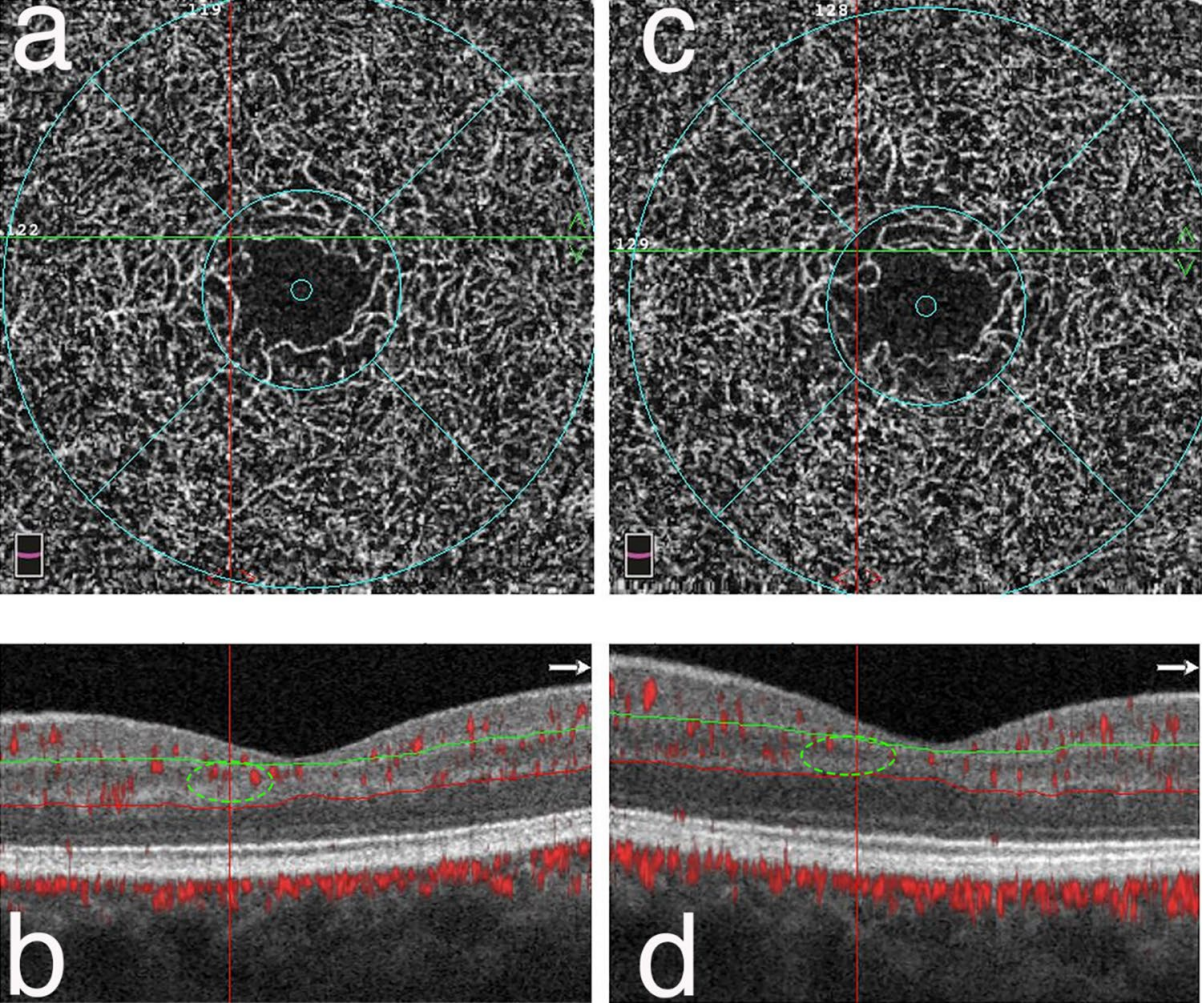
Enface OCT angiograms and structural B-scans of one DM1 patient at baseline (**a**, **b**) and after 2 years of follow-up (**c**, **d**) Enface OCT angiograms of the DCP with the corresponding structural B-scan with angio-overlay, both passing at the green and red lines. In box “c”, an area of capillary drop-out is shown in the DCP at the intersection of the two lines (red and green—box “d”) corresponding to an area with no flow signal disappearance (green circles) in B at the end of the follow-up period.

Baseline DCP PVD was slightly lower in diabetic patients than in controls (− 4.4%, *p* = 0.047) and the difference increased at 2 years (− 12.6%, *p* < 0.001). The annual linear trend was − 2.7% in diabetics vs. controls (*p* = 0.009). (Fig. 1).

In addition, the PVD of the DCP and the intermediate capillary plexus (ICP) were evaluated separately. No difference was recorded for parafoveal ICP density at individual time points (*p* > 0.1), but a statistically significant difference in the linear trend over time in diabetic patients compared to controls was recoded (− 3.2% per year, *p* = 0.001). Regarding the PVD DCP, there was no statistically significant difference in the linear trend and no statistically significant difference at any time points in diabetic patients compared to controls (*p* > 0.1 for all comparisons).

At baseline, there were not statistically significant differences in structural OCT parameters including inner limiting membrane (ILM)–inner plexiform layer (IPL) (− 4.3% thinner in diabetics versus controls, *p* = 0.273) and IPL–INL (similar at + 0.08% in diabetics versus controls, *p* = 0.708).

Furthermore, borderline significance was observed in the IPL- outer nuclear layer (ONL) (+ 4.1% thicker in diabetics vs. controls; *p* = 0.054). However, these differences were modest and not statistically significant. Moreover, the corresponding differences in annual linear trends were not statistically significant (+ 1.6%, *p* = 0.418; − 0.6%, *p* = 0.500; and − 0.8% difference, *p* = 0.531 in diabetics vs. controls, respectively).

No statistically significant association was observed on comparison of the difference between final and baseline values of structural OCT parameters with that of the OCTA parameters (DCP and SCP). In fact, the Spearman correlation was always lower than 0.25, and p-values were > 0.1 for all the pairwise correlations.

**Figure 2:**
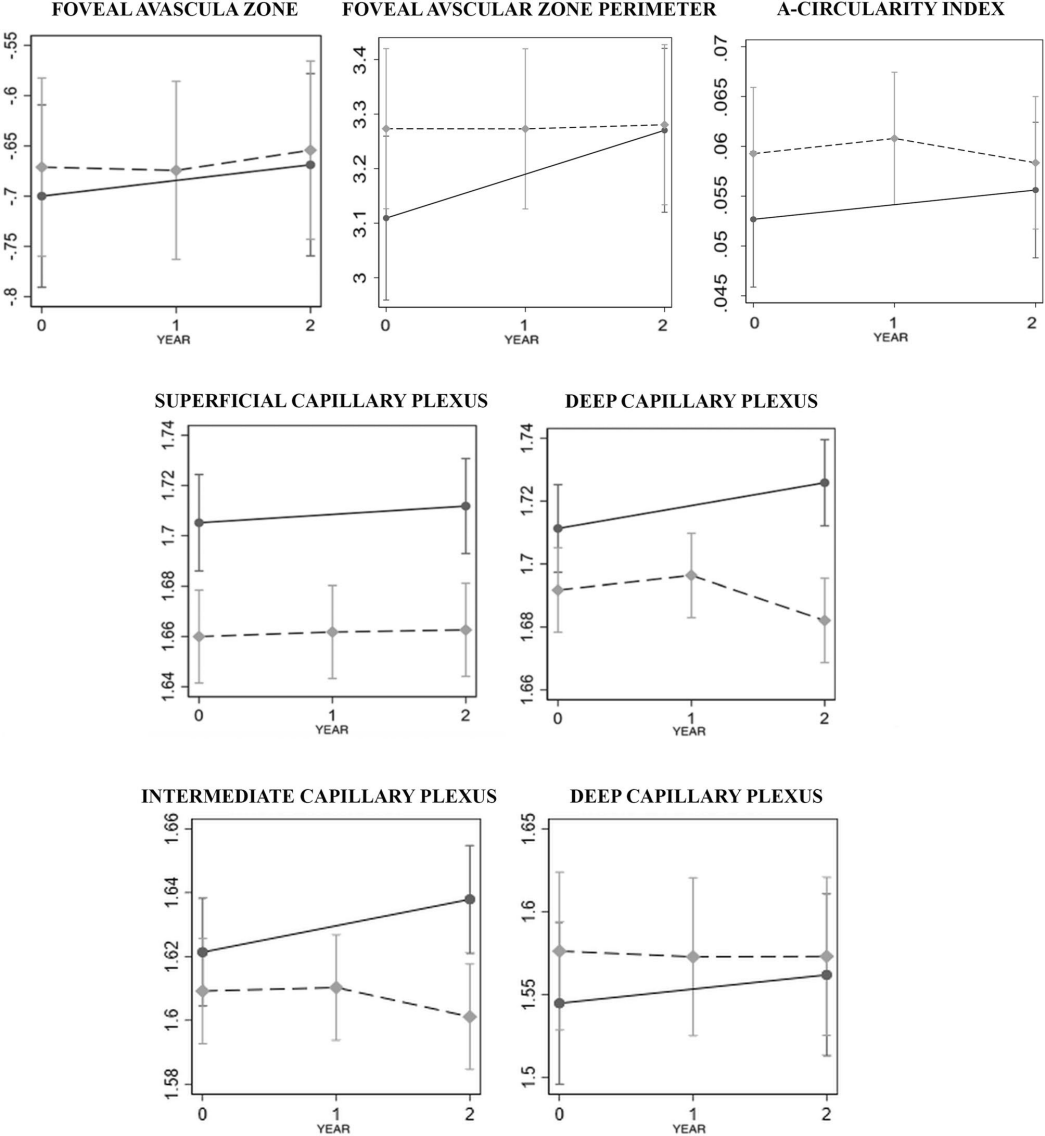
Baseline and follow-up data of each optical coherence tomography angiography OCTA parameter. Changes in the foveal avascular zone and parafoveal vessel density parameters in the superficial and deep capillary plexus, including also the intermediate capillary plexus, over time using optical coherence tomography angiography for diabetic patients (dashed line) and controls (continuous line) graphed continuously (middle line). The bottom line shows the different results when the intermediate and deep capillary plexus are analyzed separately.

**Figure 3:**
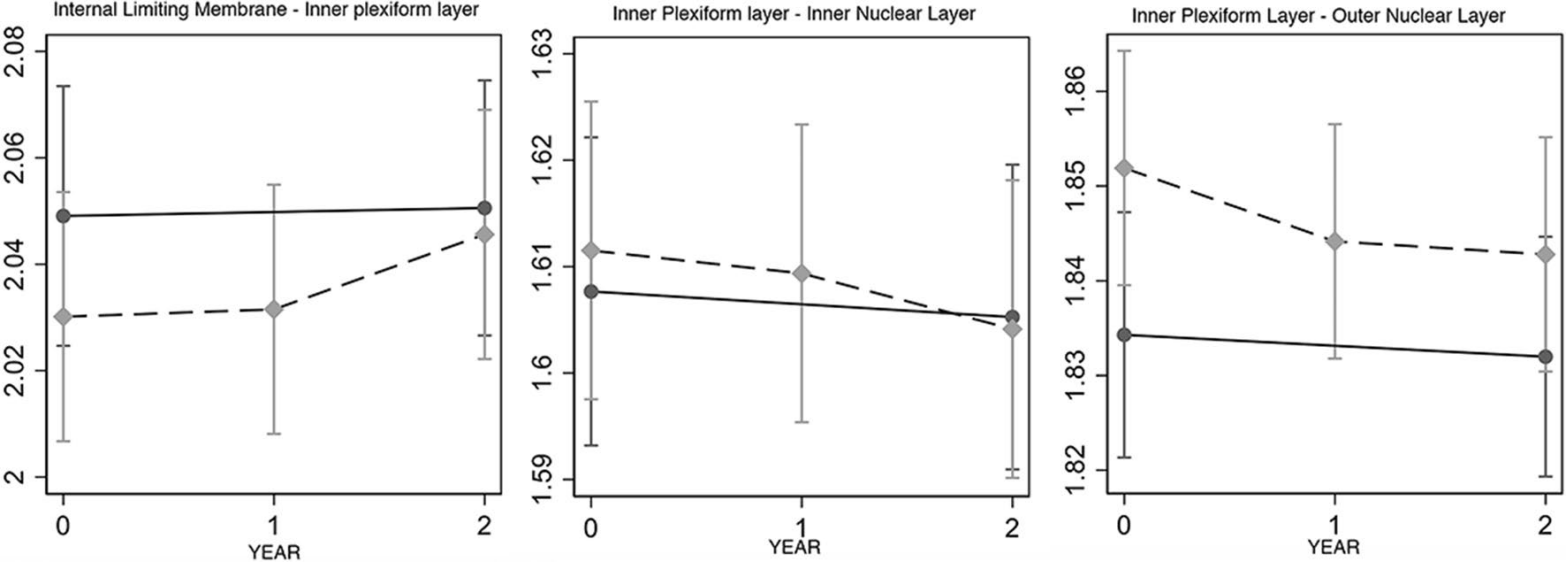
Baseline and follow-up data of each structural optical coherence tomography angiography OCTA parameter. The graphs show that despite apparent intergroup differences at baseline in structural OCT parameters, the differences were not statistically significant at the last follow up visit between diabetic patients (dashed line) and controls (continuous line).

Figures 2 and 3 present baseline and follow-up data of each structural OCTA and OCT parameters (log10 scale).

Our analyses concerning the change in all OCT/OCTA parameters and their differences between diabetics and controls did not differ when age and sex were introduced as covariates.

Baseline correlations between OCTA and structural OCT parameters in diabetic patients showed a statistically significant and moderate correlation only in the thickness of the ILM–IPL with the FAZ area (− 0.47, *p* = 0.028) and SCP PVD (0.52, *p* = 0.014). (Table 1) However, no significant correlation was found when a Bonferroni-adjusted threshold was used (*p* < 0.003 for 18 comparisons).

**Table 1:** Baseline correlations between OCTA and structural OCT parameters in diabetic patients.

	Group	ILM–IPL	IPL–INL	IPL–ONL
FAZ area	DM1	− 0.47	− 0.36	− 0.36
SCP PVD		0.52	− 0.16	− 0.26
DCP PVD		− 0.36	0.02	− 0.01

	Controls	ILM–IPL	IPL–INL	IPL–ONL
FAZ area		− 0.15	− 0.32	− 0.23
SCP PVD		0.50	0.43	0.50
DCP PVD		− 0.31	− 0.06	0.10

FAZ, foveal vascular zone; SCP, superficial capillary plexus; PVD, parafoveal vessel density; DCP, deep capillary plexus; DM1, type 1 diabetes mellitus; ILM, internal limiting membrane; IPL, inner plexiform layer; INL, inner nuclear layer; ONL, outer nuclear layer.

## Discussion

This longitudinal exploratory study of patients with DM1 showing mild clinical signs of NPDR without macular edema confirmed the fundamental role of OCTA in evaluating the progression of DR in the long term.

Our data showed a progressive decrease in PVD in the DCP in DM1 eyes compared to controls over a two-year follow-up period when the analysis of the retinal plexus was confined at two different layers (SCP and DCP). When the vascular inner retina was segmented to define three capillary plexuses (SCP, ICP and DCP), a progressive decrease in PVD in the ICP in DM1 eyes compared to controls over a two-year follow-up period was observed.

Importantly, although OCTA analysis showed that the PVD of the SCP and DCP was lower in DM1 patients compared to controls at baseline, the corresponding linear trend of blood flow diminishing over time was different between the plexuses.

Indeed, the SCP showed a linear trend of − 0.5% in diabetic versus control eyes (*p* = 0.736), while the DCP values were − 2.7% in the diabetic group versus the control group (*p* = 0.009).

These results are in line with those of several studies^37–40^ that reported an impairment of SCP and DCP vessel density in diabetic patients versus controls.

This might suggest that even if the SCP can be considered as markers of early microvascular changes in DM^40^, however the capability to detect microvascular changes over time in our group seems to be better represented by the analysis of the deeper vessels. In addition, thanks to its autoregulation, the SCP blood flow might be slightly preserved limiting the progression of vascular impairment over time^41^.

This finding not only corroborates the evidence that the deeper vessels are more susceptible to damage even in the early stages of DR, but also that its impairment might be considered as a biomarker of progression in the long-term evaluation of DR in DM1. Several factors, including distance from the larger arterioles, proximity to the high metabolic demand of the outer retina, and the complex vascular anatomical architecture may contribute to impairment of the DCP in diabetic patients^42^. Moreover, these vascular changes can be strictly correlated to the metabolic dysregulation of diabetes in this cohort since the findings were not influenced by any comorbidities including hypertension and dyslipidemia^43^.

Using OCTA, several studies have demonstrated reduced PVD at the level of the DCP in DM1 patients with no or mild DR^37,38,44–46^. These vascular alterations could precede damage in DR detectable by fundus examination.

Recently, Sun et al. provided evidence that OCTA metrics could improve the evaluation of the risk of DR progression and development of diabetic macular edema beyond traditional risk factors in eyes with the pathology^47^.

In our study, we confirmed the important role of OCTA metrics in the progression of the pathology in eyes with mild signs of DR.

In addition, because of the importance of OCTA segmentation schemes that consider the ICP separately from the SCP and DCP^40,48^, we also explored the ICP separately from the DCP in our cohort of DM1 patients.

Surprisingly, in the DCP, PVD did not show any statistically significant difference in the linear trend or difference at any time points in diabetic patients compared to controls, while, in the ICP, PVD showed a statistically significant difference in the linear trend with time in diabetic patients compared to controls (− 3.2% per year, *p* = 0.001). However, in the ICP, PVD was not statistically significant difference at individual time points (*p* > 0.1). Previous studies have shown that the ICP is particularly affected in DR, with the presence of microaneurysms and capillary loops significantly more common in this vascular layer^49–51^.

Retinal vessel density has previously been classified as a marker of disease severity in diabetic patients^52^. Onishi et al. found that parafoveal VD in all three plexuses showed a significant decrease in eyes with DR compared with healthy controls^41^. This is the first longitudinal study including only DM1 patients and we believe that these results should be interpreted with caution and further confirmed by larger scale studies. However, these findings highlighted the importance to consider the three plexuses separately in order to avoid any bias due to the ICP incorporated into the other vascular layers.

The current study found that measurement of the FAZ area and perimeter, and acircularity index did not show any statistically significant difference in either group at baseline or at the end of follow-up.

As PVD could decrease due to either diffuse capillary nonperfusion or extension of the FAZ into the parafoveal area, these results most likely represent a diffuse capillary drop out in the parafoveal area rather than enlargement of the FAZ.

Although our findings are inconsistent with those reported in previous studies^53,54^ it should be noted that the earlier studies included cases of both DM types 1 and 2. In addition, the previous studies included samples at different stages of DR; therefore, the results are not comparable.

Regarding analysis of the OCT structural parameters, statistically significant difference in the thickness of the ILM–IPL and IPL–INL slabs was not observed between diabetics and controls at baseline, whereas the IPL–ONL slab was 4.1% thicker in diabetics than in the controls (*p* = 0.054). On the contrary, the corresponding differences in annual linear trends for all aforementioned slabs were not statistically significant.

Moderate correlations were found between ILM–IPL thickness and the FAZ area (− 0.47, *p* = 0.028) in the DM1 group, and between ILM–IPL thickness and SCP PVD in both groups (0.52, *p* = 0.014).

We believe that these associations could be attributed to normal anatomic factors rather than the diabetic status, and any differences between coefficients in the two groups could possibly be due to chance.

None of the three slabs showed any statistically significant correlation with SCP and DCP changes during the study period.

In the early stage of DR, an increase in retinal thickness with subclinical macular edema principally occurs in the INL.

However, this could extend to the neighboring retinal layers due to extracellular fluid accumulation^31,32,55^. This could be the cause of increased thickness of the IPL–ONL slab observed in our cohort.

Arguably, this change may represent the onset of the vascular phase in development of diabetic macular edema or conversely “glia activation” of the Müller cells, which has been discussed in several studies^56,57^. Finally, considering the decrease in the DCP blood flow, alteration of the entire neurovascular unit cannot be excluded.

Based on these findings, it may be speculated that our cohort of DM 1 patients showed progressive vascular impairment, which was more prominent in the DCP. However, we could not identify any neurodegenerative retinal changes over time.

This observation is in contrast to that reported by Kim et al^58^. The study found a strong positive correlation between loss of macular ganglion cells/IPL and vessel density in the SCP from baseline to 24 months. However, the study included patients with DM type 2 and used different OCTA devices. Furthermore, the characteristics of the examined patients were different, considering that patients in our study were younger.

Similarly, Vujosevic et al. found that the RNFL, GCL+, and RNFL+ GCL+ (GCL++) were thicker in patients with DM1 than DM2 in 1 central mm, after adjustment for age and duration of DM. However, the difference in the GCL++ complex between DM1 and controls did not reach statistical significance^37^.

Considering these results, it could be hypothesized that vascular damage could precede the neurodegeneration (prevalent in type 2 population) in young patients with DM1. Therefore, this vascular damage might represent a biomarker for long-term follow-up of this population.

Our cohort of DM1 patients did not show peripheral neuropathy and presented overall good metabolic control during the follow-up period, as indicated by the HbA1c values. However, in a previous study, we showed that microvascular retinal abnormalities were not associated with HbA1c values, whereas early structural damage of the neuroretina in DM1 patients was related to fluctuations in glucose levels^14^. Therefore, it can be assumed that, despite an overall good metabolic control, DM1 patients in our study could have high glycemic variability, which drives the “glial activation,” and could explain the progression of microvascular impairment^59^.

The strengths of this study were its prospective study design, longitudinal follow-up, and an objective, automated quantification method (OCTA) for SCP and DCP. In addition, all image analyses were performed after adjusting for the image quality value and removal of projection artifacts.

On the contrary, the main limitation of this study was the relatively small sample size. Moreover, this study does not explain whether these results could be due to a decrease in blood flow, considering the inability of OCTA to depict slow flow, or represent ischemia. In addition, the analysis was also limited to the 3 × 3 parafoveal area.

In conclusion, our findings suggested that a decrease in PVD of DCP and more specifically of the ICP during the early stages is the most robust parameter to provide objective imaging biomarkers to monitor the clinical progression of NPDR using OCTA, while the sign of neurodegeneration could not be identified.

Early detection of the progression of vascular damage and future therapy might be useful to avoid progression to functional as well as morphological retinal impairment due to development of diabetic macular edema.

These findings can also improve our understanding of the pathophysiology of DR by noninvasive measurements.

Indeed, microvascular impairment of the causing initial hypoxia might represent the tipping point that compromises the large difference in oxygen tension between retinal arteries and veins at the level of smaller vessels. This pathological microvascular abnormality may cause early damage to the retinal vascular endothelial cells and promote the passage to the more severe stage of chronic hypoxia and its consequences over time.

## Materials and methods

This longitudinal case–control exploratory clinical study was performed according to the Declaration of Helsinki, and informed consent was obtained from all study participants. The study was formally approved by the Institutional Review Board of Fondazione Bietti - IRCCS, Rome, Italy (protocol n° Ret03/2016/ December 22, 2016).

The diagnosis of DR was based on a comprehensive medical and ophthalmic history and full ophthalmologic examination, including best-corrected visual acuity, external slit lamp, and fundus examination. Two experienced examiners (MP and FS) identified the eyes with mild NPDR based on the analysis of color fundus photographs, according to the modified Early Treatment Diabetic Retinopathy Study (ETDRS) retinopathy severity scale and analyzed the B-scan OCT images to exclude the presence of edema^19,60^.

Patients with DM1 and NPDR and healthy control subjects were included. Only data from the right eyes were analyzed. Enrollment criteria included a diagnosis of DM1 made at least before 1 year and age ≥ 18 years, with NPDR.

Exclusion criteria included poor metabolic control (HbA1c > 9%), evidence of macular edema on clinical examination or OCT macular B-scan, high myopia (< 6.00 diopters), lens opacity that could affect imaging, and history of any other retinal disease or intraocular surgery. Furthermore, subjects with any systemic disease, including hypertension, hyperlipidemia, cardiovascular or cerebrovascular disorders, malignant tumors, renal impairment, or kidney transplantation were excluded.

### OCTA image collection and analysis

Images were obtained using the ANGIOVUE OCTA software of the commercially available RTVue XR spectral domain-OCT device (Optovue, Inc., Fremont, CA, USA). This instrument has an A-scan rate of 70,000 scans per second and uses a light source centered at 840 nm and a bandwidth of 45 nm. A split-spectrum amplitude-decorrelation angiography (SSADA) algorithm, explained in detail elsewhere (Jia et al. 2012), was used to detect erythrocyte movement and depict blood flow in a 3 × 3 mm scanning area centered on the fovea.

Enface OCT angiograms were segmented to define the SCP and DCP using the built-in software segmentation algorithm. The SCP slab was obtained from the ILM to the IPL/INL (9 μm above). The DCP slab was obtained from the IPL–INL junction (9 μm above) to the outer plexiform layer (OPL)/ ONL junction (9 μm below).

In addition, we segmented the DCP into intermediate capillary plexus (ICP) and DCP by manually adjusting segmentation boundaries^48^. The ICP boundaries were set between 9 mm above the IPL–INL junction and 6 mm below the INL–OPL junction, thus including parts of the IPL and OPL and all the INL to record the ICP projection that is located in the IPL. The DCP boundaries were set between 6 mm below the INL–OPL junction and 9 mm below the OPL–ONL junction, thus including the OPL.

The ‘parafovea’ was defined as the area within an annulus centered on the fovea with inner and outer ring diameters of 1 and 2.5 mm, respectively. PVD, defined as the percentage of the total area occupied by the vessels and microvasculature, was quantified in the SCP and DCP. To calculate vessel density, the ANGIOVUE analytics software was used to extract a binary image of the blood vessels from the grayscale OCTA image and subsequently calculate the percentage of pixels with a flow signal above the preset decorrelation threshold in the defined region.

We used the 3D projection artifact removal (PAR) tool by Optovue to acquire 3 × 3 mm images centered on the fovea^61^. This algorithm uses data from the volume of OCT as well as OCTA to separate the OCTA signal from projection artifacts considering the intensity profiles of OCTA and OCT anterior to and at the voxel of interest^62^.

Unlike conventional PAR algorithms, 3D PAR preserves the signal strength to better show real vasculature.

FAZ area, FAZ perimeter (mm), and a-circularity index FAZ perimeter (mm) were measured using the software function.

Patients and controls underwent three sets of OCTA imaging at baseline and at each follow-up visit. The average values were considered for the analysis.

The evaluation in this study focused on the baseline, one-year (12 months), and the two-year follow-up (24 months) visits. Two readers (MP and FS) verified the correctness of the OCTA image segmentation. Image quality was ascertained by excluding images with a quality index of < 5 and the presence of motion artifacts. The scans were repeated in patients with poor images.

### OCT image collection and analysis

Thickness of the retinal layers was measured on the structural map corresponding to the 3 × 3 mm OCTA map obtained simultaneously with the vessel density map. The OCT software automatically allows measurement of the thickness of individual retinal layers within 10 predefined slabs from the ILM to the Bruch’s membrane. In this study, three predefined slabs were considered: the ILM–IPL, IPL–INL, and IPL–ONL.

**Figure 4:**
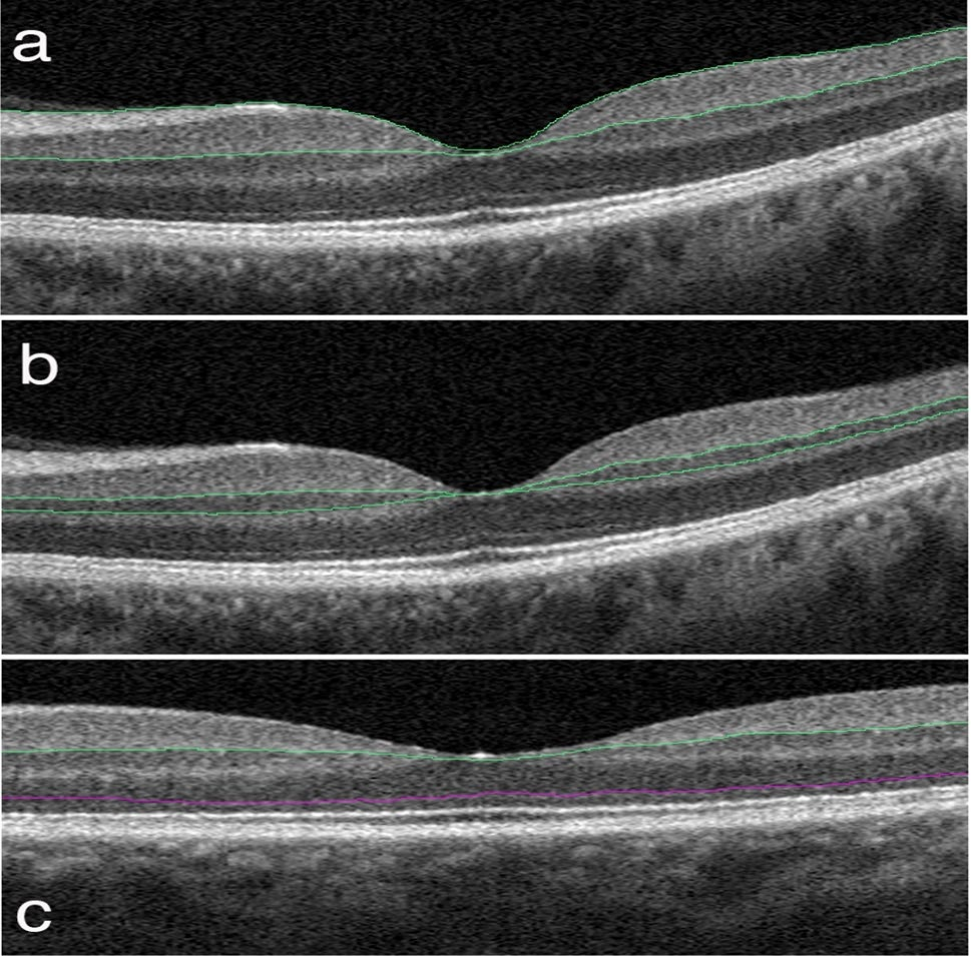
Structural OCT slabs. The segmentations of the three slabs considered in the analysis are as follows: (**a**) ILM to the IPL, (**b**) IPL to the INL, and (**c**) IPL to the ONL.

The SCP therefore included the nerve fiber layer (NFL), GCL, and the major part of the innermost portion of the IPL, whereas the DCP included the outer portion of the IPL, INL, OPL, and inner portion of the ONL. (Fig. 4).

### Statistics

Data are expressed as mean ± SD. Data were normally distributed as expressed by means of the Shapiro–Wilk test and independent, two-tailed Student’s t-tests were used to compare the two groups.

We used linear mixed models with subjects as random effects to account for correlated longitudinal data. All analyses were corrected using the overall quality index. Pearson correlations were used to investigate the relationship between the change in OCT and OCTA parameters over time (log10-transformed).

All statistical analyses were conducted using Stata 15.1 software (StataCorp, College Station, TX, USA).

## Data Availability

The data used to support the findings of this study are available from the corresponding author upon request.
