# Author Correction: Microvascular impairment as a biomarker of diabetic retinopathy progression in the long-term follow up in type 1 diabetes

**DOI:** 10.1038/s41598-021-84931-1

**Published:** 2021-03-02

**Authors:** Fabio Scarinci, Fabiana Picconi, Gianni Virgili, Monica Varano, Paola Giorno, Simona Frontoni, Mariacristina Parravano

**Affiliations:** 1 Department of Ophthalmology, IRCCS - Fondazione Bietti, Rome, Italy; 2 Unit of Endocrinology, Diabetes and Metabolism, S. Giovanni Calibita Fatebenefratelli Hospital, Department of Systems Medicine, University of Rome Tor Vergata, Rome, Italy; 3 Department of Neurosciences, Psychology, Drug Research and Child Health (NEUROFARBA), University of Firenze and AOU Careggi, Florence, Italy

Correction to: *Scientific Reports* 10.1038/s41598-020-75416-8, published online 26 October 2020

The original version of this Article contained an error in Affiliation 1, which was incorrectly given as ‘Department of Ophthalmology, G.B. Bietti Eye Foundation-IRCCS, Via Livenza, 3, 00198 Rome, Italy’. The correct affiliation is listed below:

Department of Ophthalmology, IRCCS - Fondazione Bietti, Rome, Italy

This error has now been corrected in the HTML and PDF versions of the Article.

